# Effect of L-carnitine on in vitro developmental rate, the zona pellucida and hatching of blastocysts and their cell numbers in mouse embryos

**Published:** 2016-10

**Authors:** Nasrin Khanmohammadi, Mansoureh Movahedin, Manouchehr Safari, Hamid Reza Sameni, Behpour Yousefi, Behnaz Jafari, Sam Zarbakhsh

**Affiliations:** 1 *Research Center of Nervous System Stem Cells, Department of Anatomy, Faculty of Medicine, Semnan University of Medical Sciences, Semnan, Iran.*; 2 *Anatomical Sciences Department, Faculty of Medicine, Tarbiat Modares University, Tehran, Iran.*

**Keywords:** *L-carnitine*, *Embryo culture*, *Zona pellucida*, *Blastocyst inner cell mass*

## Abstract

L-carnitine (LC) is an antioxidant with the ability to promote the growth in vitro embryo.

**Objective:**

The goal was to evaluate the effect of LC on some indicators of embryo development and blastocyst quality including zona pellucid (ZP) thickness, the hatching of blastocysts and their cell numbers.

**Materials and Methods:**

Mouse embryos were randomly divided into five groups and incubated with different concentrations of LC (I; 0, II; 0.5, III; 1, IV; 2 and V; 4 mg/ml) from 2-cell to hatched blastocyst. The percentage of blastocysts and hatched blastocysts was calculated. Blastocysts ZP thickness was measured and the number of blastocyst cells was counted using Hoechst and propidium iodide (PI) staining.

**Results:**

The results showed concentration of 0.5 mg/ml of LC had an antioxidant effect as in this group, the percentage of blastocysts and hatched blactocysts (p=0.01), the ZP thickness (p=0.00) and the number of blastocyst inner cell mass were significantly more favorable than the control group (p=0.03); and concentration of 4 mg/ml of LC had a toxic effect on embryo development and blastocyst quality (p=0.00).

**Conclusion:**

The results suggest that LC may increase the number of blastocyst cells, which probably helps to expand the blastocyst and thinning of the ZP thickness and, therefore, creating a successful hatching for implantation.

## Introduction

Infertility is an important problem in developed countries. Recently using assisted reproductive technology (ART) has emerged more favorable status (1). ART was confirmed more than 30 years ago and it is estimated that about 3.9% of all births are the result of this method. One of the most common ART procedures is embryo in vitro culture (IVC) (2, 3). Embryo culture gives time to show its potential and has become a valuable tool for embryo selection. Observation of development in a group of embryos and their selection for transfer to the uterus is based on blastocyst morphology (4). 

To get more blastocysts with high implantation potential, optimization of culture medium may be necessary (5). It is well known that in vitro embryo development can be damaged by several stressors, such as visible light or high oxygen concentration (6). Several studies have indicated that higher levels of reactive oxygen species (ROS) produced through IVC can reduce the rate of fertilization, embryo development and pregnancy (7, 8). 

Moreover, blastocyst formation during ART is not desirable, and the use of antioxidants may improve it (9). It is important to keep embryos in culture medium from oxidative stress and for this aim, antioxidants are a valuable candidate (10, 11). L-carnitine (LC) is a water-soluble antioxidant which known to play an essential role in fatty acid metabolism (12-14). Several articles have shown that LC plays a pivotal role in β-oxidation by transporting fatty acid into mitochondria for ATP production, which can raise embryo development and improve the blastocyst formation rate (9, 15). In addition, LC has free radica-scavenging activity and inhibits lipid peroxidation, thereby protects against damage induced by hydrogen peroxide (H_2_O_2_) (9).

By observing embryo quality, the likelihood of successful implantation and pregnancy can be predicted. Despite the fact that many embryos have developed well, they have failed in implantation, and this is probably associated with hatching problems (16). Hatching is an exigent process where blastocyst escapes through the zona pellucida (ZP) before implantation. ZP Thickness is an important indicator for successful hatching and implantation of transferred embryos (17). ZP Elasticity and thinning are essential for hatching process, which can be adversely affected by increasing maternal age and embryo culture conditions. A thick ZP may be associated with low quality of embryos (18). Our hypothesis was that ZP thickness and blastocyst hatching might be affected by improving the culture media with LC. 

The first discernible cell lineage during the mouse embryo development is organization of precursor cell populations of inner cell mass (ICM) and trophoectoderm (TE) (19). Du *et al* suggest that the number of cells in the blastocysts may be an important indicator for embryo quality and implantation (20). Abdelrazik *et al* reported that LC could improve the blastocyst formation rate in mice (9). Currently, there is insufficient document regarding the effect of LC on morphological parameters of blastocyst. 

In this study, we evaluated the effect of different concentrations of LC on some indicators of embryo development and blastocyst quality in vitro including ZP thickness, hatching of blastocysts and their cell numbers.

## Materials and methods

Mice obtained from Tehran Pasteur Institute (NMRI, female: 6-8 wks old; and male: 10 wks old), were kept in an air-conditioned room under controlled temperature (25±2^o^C) and controlled light (12 hr light/dark), with free access to food and water. All animal protocols were approved by the Semnan University of Medical Sciences animal Ethics Committee and enforced accordance with university guidelines.

**Recovery of embryos**

Female mice were superovulated by a 10 IU intra peritoneal injection of pregnant mare's serum gonadotropin (PMSG) (Sigma Aldrich, China) followed by injection of human chorionic gonadotropin (hCG) (Sigma Aldrich, China) (21). They were mated overnight with males and the mating was emphasized by the presence of vaginal plug on the morning after hCG injection. The 2-cell embryos were flushed from the oviduct at 48-50 hr after hCG injection and washed in human tubal fluid (HTF) medium containing HEPES (Sigma Aldrich, China). A total of 450 two-cell embryos were used in this study (90 embryos in each group).

**Embryo culture**

2-cell embryos were transferred into HTF medium (supplemented with 10% human serum albumin) (Sigma Aldrich, China) and were randomly divided into five groups with different concentrations of LC (I; 0.0, II; 0.5, III; 1, IV; 2 and V; 4 mg/ml) (Sigma Aldrich, China) (9). There was no LC in the control group. In all groups, 10 embryos were situated in a drop of 20 µl of HTF medium under mineral oil (Sigma Aldrich, USA) in a 35 mm Petri dish (Jet Biofil, Canada) and were incubated at 37^o^C with 95% humidity and 5% CO_2 _(22). In 120 hrs after incubation onset, the rate of development to hatched blastocysts was assessed as the percentage of 4-cell, 8-cell, morula, blastocyst and hatched blastocyst stages (23).

**Measurement of ZP thickness**

ZP thickness was randomly measured in early and full blastocyst stages. Measurement was taken from images using an inverted microscope (Nikon, Eclipse Ti-U, Japan) and motic images plus 2.0 software. The thickness of each ZP was measured at 3 points (24).

**Differential staining of blastocysts**

Expanded blastocysts were randomly selected for cell counting analysis. The embryos were treated with 0.1 mg/ml propidium iodide (PI) (Sigma Aldrich, China) and 1% Triton X-100 at 37^o^C for 10 sec and were instantly transferred into 25 µg/ml bisbenzimide (Hoechst 33258) (Sigma Aldrich, USA) and stored at 4^o^C overnight. The embryos were then mounted on glass slides in glycerol droplets and were observed under an inverted fluorescent microscope (Motic, AE31, Spain). ICM nuclei labeled with Hoechst (blue) and TE nuclei labeled with PI (red). The number of ICM, TE and total embryonic cells was counted (25).

**Statistical analysis**

Comparison of the percentage of embryonic developmental stages between the experimental groups and the control group was analyzed by ^2^ test. The ZP thickness and the blastocyst cell count were analyzed by one-way analysis of variance (ANOVA) followed by the Tukey test as mean±SD. A difference with p<0.05 was considered statistically significant.

## Results

**Findings of developmental rate of embryos**

**Figure 1:**
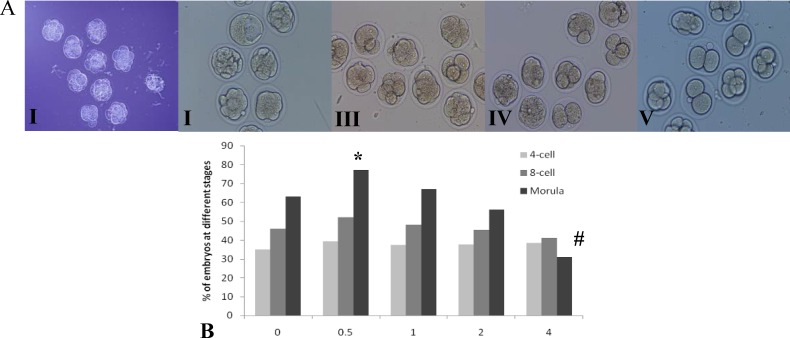
A. The cleavage stages at 48 hours culture following L-carnitine exposition (×200). (I) Control group, the embryos are at 8-cell and morula stages, (II) 0.5 mg/ml, the embryos are at morula stage and in one of them blastocoele is composed, (III) 1 mg/ml, the embryos are at 4-cell and 8-cell stages, (IV) 2 mg/ml, there is a slight delay in development as the embryos are at 2-cell, 4-cell and 8-cell stages, (V) 4 mg/ml, there is a delay in development as the embryos are at 2-cell and 4-cell stages. B. The results of in vitro development of 2-cell embryos to morula stage in different concentrations of L-carnitine.

^* ^Favorable effect of L-carnitine versus control group (p<0.05).

^# ^Adverse effect of L-carnitine versus control group (p<0.05).

A total of 450 embryos were used to evaluate the effect of different concentrations of LC on in vitro development of 2-cell embryos to hatched blastocysts. In the early stages of embryo culture, no statistically significant difference was observed in the percentage of 4-cell and 8-cell stages between the experimental groups and the control group (p=0.67). The percentage of embryos that reached to morula stage in the 0.5 mg/ml of LC group was significantly higher than the control group (p=0.01) and there was no statistically significant difference between 1 and 2 mg/ml of LC groups than the control group (p=0.23). In addition, the percentage of morula in the 4 mg/ml of LC group was significantly lower than the control group (p=0.00) (Figure 1).

**Figure 2:**
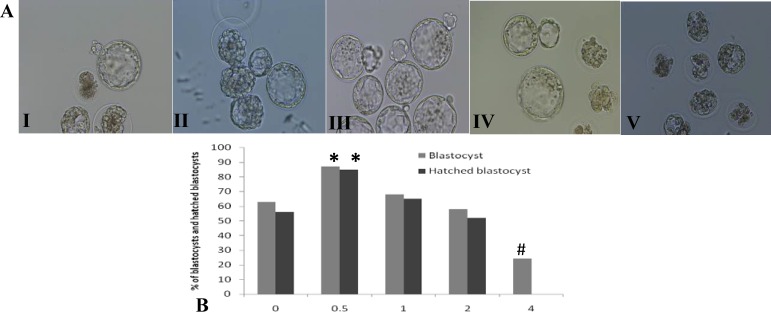
A. Blastocysts and hatched blastocysts in different concentrations of L-carnitine (×200). (I) Control group, some of the blastocysts have grown and some are dead, (II) 0.5 mg/ml, the blastocysts are hatching, (III) 1 mg/ml, the blastocysts have grown and have started to hatch, (IV) 2 mg/ml, some of the blastocysts have grown and some of them have developmental delay or dead, (V) 4 mg/ml, all embryos are dead. B. The results of the percentage of blastocysts and hatched blastocysts in different concentrations of L-carnitine

^*^ Favorable effect of L-carnitine vs. control group (p<0.05).

^#^ Adverse effect of L-carnitine vs. control group (p<0.05).

In the late stages of embryo culture, the results of percentage of embryos that reached to blastocyst and hatched blastocyst stages were almost similar to the results of the morula stage (p=0.01), as revealed an antioxidant effect of 0.5 mg/ml of LC and a toxic effect of 4 mg/ml of LC on mouse embryo development. In the 4 mg/ml of LC group, most of the blastocysts died and none were hatched (Figure 2). 

**Findings of ZP thickness**

**Figure 3:**
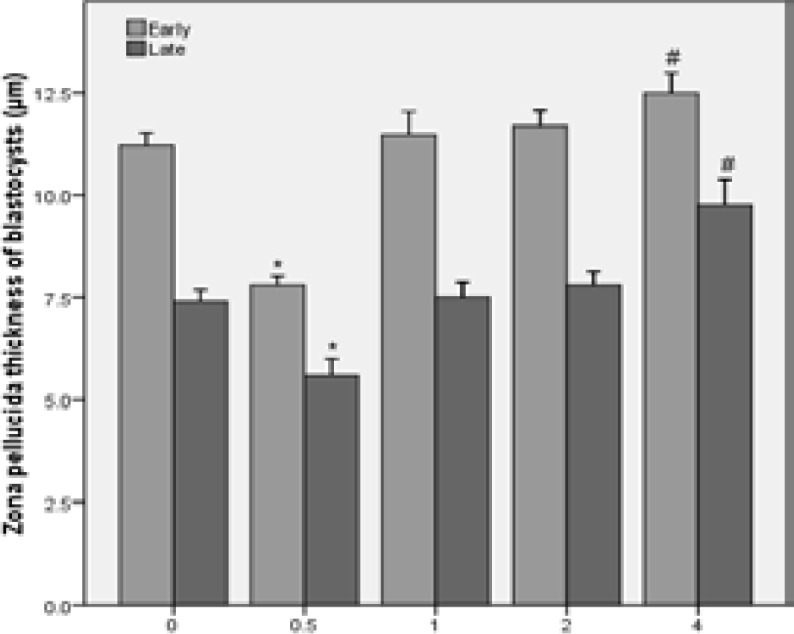
The results of zona pellucida thickness of early and full blastocysts in different concentrations of L-carnitine.

^*^ Favorable effect of L-carnitine vs. control group (p<0.05).

^#^ Adverse effect of L-carnitine vs. control group (p<0.05).

Thinning of ZP occurs during the time interval between early and full blastocyst. The results showed that the ZP thickness of early and full blastocysts after incubation of embryos with 0.5 mg/ml of LC was significantly thinner than the control group (p=0.00). There was no statistically significant difference between 1 and 2 mg/ml of LC groups than the control group and the ZP thickness in the 4 mg/ml of LC group was significantly thicker than the control group (p=0.00) (Figure 3).

**Findings of blastocyst cell count**

**Figure 4:**
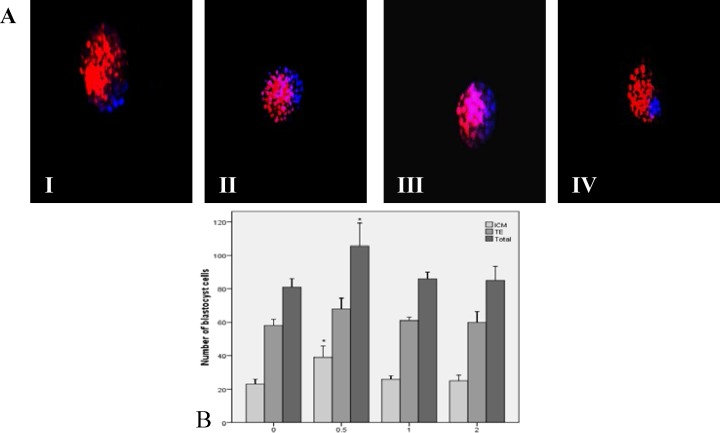
A. Differential staining of blastocysts in different concentrations of L-carnitine (×200); (I) Control group, the average number of inner cell mass (ICM) is moderate, (II) 0.5 mg/ml, the average number of ICM is higher than the control group, (III) 1 mg/ml, the average number of ICM is almost the same as the control group, (IV) 2 mg/ml, the average number of ICM is almost the same as the control group too. B. The results of blastocyst cell numbers in different concentrations of L-carnitine.

^*^ Favorable effect of L-carnitine versus control group (p<0.05).

Expanded blastocysts were stained with Hoechst and PI. The ICM, TE and total cell numbers were counted. The number of ICM and the total cell numbers in the 0.5 mg/ml of LC group was significantly higher than the control group (p=0.03). There was no statistically significant difference between 1 and 2 mg/ml of LC groups than the control group (p=0.87). There was no statistically significant difference in the TE cell numbers between the experimental groups and the control group (p=0.85). Since the blastocysts in the medium with 4 mg/ml of LC were not expanded, this staining was not performed in this group (Figure 4).

## Discussion

In this study, we added different concentrations of LC into embryo culture medium and evaluated some indicators of embryo development and blastocyst quality including the ZP thickness, the hatching rate and the number of blastocyst cells. Overall, the results showed the concentration of 0.5 mg/ml had an antioxidant effect and the concentration of 4 mg/ml had a toxic effect compared with the control group. 

Development of a suitable culture system is necessary for successful production of embryo in vitro (26). During this process, oxidative stress is one of the detrimental agents on fertilization and embryo quality (11). Since oxidative stress during embryo culture has been known as one of the main factors responsible for poor quality of embryos, it has been suggested that environmental factors such as increasing free radical scavengers and reducing the oxygen tension are essential for improving the fertility potential in ART (26, 27). 

During embryo culture, oxidative stress pressure may be regulated by adding antioxidants into culture media (10, 28). LC is an antioxidant that its antioxidant capacity has been shown in other cell types, including lymphoma cells and skeletal muscles (29, 30). Moreover, the useful effect of LC during in vitro maturation (IVM) and IVC has been reported in many mammalian species. In mice, it has been shown that adding 1mM of LC into IVM medium enhances β-oxidation, nuclear maturation and blastocyst development (31, 32). We showed that 0.5 mg/ml of LC improves development of 2-cell embryos to hatched blastocyst stage. These results are in accordance with other reports (9, 14). This positive effect is probably due to two reasons: 1. Enhancing mitochondrial lipid metabolism and 2. Act as an antioxidant to reduce oxidative stress (9, 32). 

Several studies have shown that the number of blastocyst inner cell mass is indicative of embryo quality and is crucial for a successful implantation (25, 33, 34). We showed that favorable dose of LC increased the ICM. These results are in agreement with the other studies (14, 15, 31). The number of ICM is important for appropriate implantation, and several studies have reported that a reduction in the number of ICM during development can lead to reduced embryonic viability (25, 35, 36). Blastocoel fluid has H_2_O_2_ which is cytotoxic and induces apoptosis in ICM of blastocyst (37). The antioxidant effect of LC could probably increase intracellular glutathione level and since glutathione is involved in removal of H_2_O_2_, ICM would not become apoptosis (38). LC plays a key role in β-oxidation of long-chain fatty acids in mitochondria to generate cellular ATP, it can possibly increase the number of ICM by increasing ATP (14).

Embryonic cells are surrounded by ZP. Shiloh *et al* showed that ZP thickness of embryos isolated from cigarette smokers was thicker than non-smokers which indicated environmental factors could affect the thickness of ZP (39). Moreover, other reports have suggested ZP thickness variation is a dependable marker to select the best frozen and thawed embryos for transfer (40). ZP thickness depends on inherent properties of human embryo to produce the lytic factors required for ZP thinning. Indeed, expression of murine implantation proteinase genes (ISP1 and ISP2) has been indicated recently throughout all stages of preimplantation development from zygote to blastocyst, but synthesis of their products (ISP1-ISP2 enzyme complex) was detected only in early blastocysts (17, 41, 42). 

Therefore it has been suggested that the embryonic ISP enzymes play a vital role in ZP lysis, embryo hatching, and implantation (17). We showed adding 0.5 mg/ml of LC into embryo culture medium could cause thinning of the ZP thickness and in the blastocysts with thinner ZP, hatching rate was significantly higher. These results are in agreement with other studies that the thinner ZP is essential for hatching process (43). In vitro culture of mammalian embryos changes the characteristics of their ZP; as in vitro derived embryos have thicker ZP, specially the inner layer of ZP (44). There is not enough information about the effect of antioxidants on ZP thickness of blastocyst, while the thickness of ZP is one of the most important factors for hatching and implantation (45). Hatching and implantation occur in high-quality blastocysts which have expanded enough (46). 

It seems blastocyst expansion depends on the number of blastocyst cells and an increase in these cells leads to thinning of the ZP, hatching blastocyst and finally implantation (43). More research is necessary to clarify the molecular mechanisms underlying LC function on development and quality of embryos.

## Conclusion

The results suggest that the concentration of 0.5 mg/ml of LC may have an antioxidant effect and the concentration of 4 mg/ml of LC may have a toxic effect on in vitro embryo development. Antioxidant effect of LC can probably increase the number of embryonic cells, which helps to expand the blastocyst and thinning of the ZP thickness and, therefore, creating a successful hatching for implantation.
